# The defining characteristics of pasture-based systems in temperate regions

**DOI:** 10.3168/jdsc.2025-0981

**Published:** 2026-05-01

**Authors:** B. Horan, S. Stirling, R. Bryant, L. Delaby

**Affiliations:** 1 Animal and Grassland Research and Innovation Centre, Teagasc Moorepark, Fermoy, Co. Cork, Ireland; 2 Instituto Nacional de Investigación Agropecuaria (INIA), Programa Nacional de Investigación en Producción de Leche, Estación Experimental INIA La Estanzuela, 39173 Colonia, Uruguay; 3 Lincoln University, Faculty of Agriculture and Life Sciences, Lincoln 7647, New Zealand; 4 INRAE, L'Institut Agro, Physiologie, Environnement et Génétique pour l'Animal et les Systèmes d'Elevage, F-35590 Saint-Gilles, France

## Abstract

Pasture is an important source of feed for dairy and beef cattle in many parts of the world, but its predominant use in some dairy production systems such as those traditional to Western Europe, South America, and Oceania is relatively unique, with only 10% to 15% of global dairy products derived from such systems. Recently, pasture-fed dairy products have commanded premium international market prices as consumers perceive them to be superior to products from conventional confinement-based systems. There is, however, no international standard that defines the term grass/pasture fed and diverse definitions apply across different countries. To provide a practical definition of the dietary characteristics of pasture-based systems, we reviewed the literature from recent full-lactation farm systems experiments which have reported the proportion of grazed pasture in the total animal diet, in addition to grazing platform stocking rate (SR), grazing days per cow and per hectare without forage supplements, and concentrate supplementation (kg DM/cow). The final database contained 56 experimental comparisons published between 2008 and 2025. Based on the data, the proportion of grazed pasture in the total animal diet was described using linear regression according to 3 factors, namely, the number of full grazing days achieved without forage supplementation (no. of days), the grazing SR (cows/ha) on the grazing area, and the total level of concentrate supplementation (kg DM/cow). The results of the linear regression indicate that these 3 parameters explain a large part of the variation in grazed pasture proportion. Based on the data reported in these studies, we propose that a pasture-based dairy system can be defined as a system where the total diet of the dairy cow during the combined lactating and nonlactating period (equivalent to one complete year) contains in excess of 50% of grazed pasture on a DM basis, and where less than 15% of the total animal feed requirements are supplied from concentrate supplements (equivalent to approximately 1,000 kg DM/cow).

## Highlights

• Within a pasture-based system, the total diet of the dairy cow should contain >50% grazed pasture on a DM basis.
• +100 grazing days/ha without supplement = +3% pasture in the diet.
• −500 kg DM concentrate supplementation per cow = +4% pasture in the diet.

## Graphical Abstract

A pasture-based dairy system can be defined as a dairy production system where the total diet of the dairy cow during the combined lactating and nonlactating period (equivalent to 1 complete year) contains a minimum of 50% of grazed pasture on a dry matter (DM) basis. Such systems are based on a large number of pasture-only grazing days per hectare on the grazing platform (>500 days) without other conserved forage supplements and with less than 15% of total animal feed requirements (equivalent to approximately 1,000 kg DM/cow) supplied from concentrate supplements.

**Figure:**
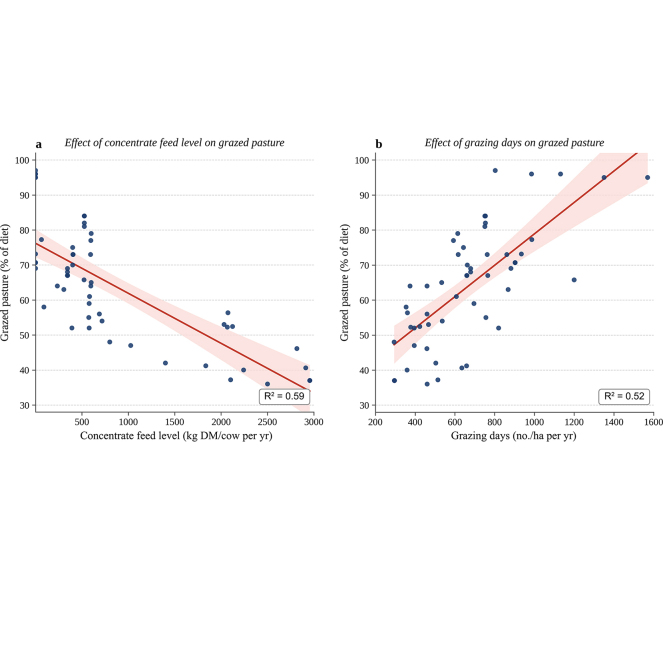

Across the world, grasslands contribute significantly to global agricultural production, covering more than 40% of the earth land surface (excluding Greenland and Antarctica) with a large diversity of vegetation (White et al., 2000). Although grazing land is the single largest land-use category, the intensity of land use varies considerably within and among different land-use types and regions with approximately 10% of the total ice-free land surface managed intensively (Erb et al., 2016). The term “grassland,” which is synonymous with pastureland, is defined by Allen et al. (2011) as land with an imposed grazing-land ecosystem of managed pastures comprising grasses, legumes, and other herbaceous species vegetation that are directly grazed by livestock. A large part of the total grassland area is composed of native or natural grassland such as the savanna in Africa, the pampa in South America, shrub land and steppes in Oceania and Asia, and tundra in Europe. Indeed, intensive and semi-intensive grasslands represent only a minor component (2%) of total land use (IPCC, 2019). In addition to the efficient conversion of human indigestible feed to high quality human utilizable nutrients (Peyraud, 2017; Mottet et al., 2017), there is also a growing appreciation for grasslands in ecosystem equilibrium, including as an important biological filter that reduces nutrient and chemical losses to surface and ground water, conserves soils, and supports enhanced biodiversity and carbon storage (O'Mara, 2012; Soussana and Lemaire, 2014; Plantureux et al., 2016). Indeed, in many temperate regions with traditional pasture-based systems, improving the efficiency of grasslands is considered as a key opportunity to develop more resilient and climate-friendly farming systems for the future (Peyraud et al., 2010). From an economic perspective, grazed pasture provides a relatively inexpensive and uniquely nutritious feed source for milk production and, consequently, farm profitability is closely related to the quantity of pasture consumed (t DM/ha; Hanrahan et al., 2018; Neal and Roche, 2019). At the same time, Dillon et al. (2008) has also shown that a strong negative relationship exists between the total costs of milk production and proportion of grazed pasture in a dairy cow's diet. Nevertheless, despite their widely acknowledged influence on the economic performance of grazing based systems, accurately quantifying both pasture utilization (t DM/ha) and dietary grazed pasture proportion (% DM) is challenging for commercial farmers in practice, necessitating the development of more tangible practical proxy measures to assess and improve the efficiency of grazing systems.

Although pasture is an important feed source for livestock worldwide, its predominant use in dairy production systems in Western Europe, South America, and Oceania is unique with more than 50% of total annual animal feed requirements on a DM basis supplied from grazed pasture. Indeed, O'Brien et al. (2018) has suggested that only 10% to 15% of global dairy products are derived from such systems. In recent decades, growth in global dairy consumption has typically led to larger and more intensive dairy farms with animals housed and typically fed ensiled forages and grains to increase milk production (Alvarez and del Corral, 2010; Winsten et al., 2010). At the same time, increasing consumer concerns over conventional confinement production practices has led to the development of a range of food products that differentiate based on the use of grazed pasture. Today, pasture-based or pasture-fed dairy products command premium market prices (McCluskey et al., 2009; Xue et al., 2010; Wang et al., 2023). Such premiums are recognition of the perceived “naturalness” of the foods produced from pasture, the appealing landscapes of grazing ruminants outdoors, and the superior welfare of farmed animals within such systems (Moscovici Joubran et al., 2021; McGuinness et al., 2022). There is, however, no agreed international standard that defines the term pasture fed, and diverse definitions and standards (such as the amount of time animals spend outdoors or the proportion of the total fresh or DM dietary content supplied from pasture or pasture silage and other grown feedstuffs, and so on) apply across different countries. Consequently, labeling of pasture-fed products is often ambiguous and confusing for consumers (Moscovici Joubran et al., 2021). Evidence suggests that consumers have little knowledge about pasture-fed dairy practices (Schiano et al., 2020) and there is an inclination to presume that all dairy products originate from such sources (McCluskey et al., 2009; Stampa et al., 2020). The objective of this article is, first, to describe the defining characteristics of pasture-based dairy systems and, second, in the particular case of temperate grazing low and midland grazing systems, to provide a practical definition of the dietary characteristics of such systems.

Among dairy production systems, a grazed pasture-based model is peculiar in design, due to its high reliance on climatic conditions for the production of perishable feed and compactly calved and highly selected grazing animals for the autonomous management of feed quality and utilization. In export-oriented dairy industries that typically experience comparably greater milk price volatility, pasture-based dairy systems with a high grazed pasture proportion of total feed requirements can achieve lower milk production costs and greater economic margins (per cow and per liter) across a wide range of milk prices (Dillon et al., 2008; Ramsbottom et al., 2015; Neal and Roche, 2019). The comparative advantage of such systems arises from the possibility of supplying a significant proportion of the total feed required from grazed pasture, with minimal expensive supplement costs. Dillon et al. (2008) has also illustrated that, although the total costs of milk production are reduced as the proportion of grazed pasture in dairy cow diets increases, it is only when most of the diet is composed of grazed pasture that substantial cost savings can be achieved. At the same time, recent commercial farm financial performance evaluations within grazing systems (Ramsbottom et al., 2015; Fariña and Chilibroste, 2019; Neal and Roche, 2019) reveal that, within the range of commercial farm systems operated, net farm profitability per hectare is maximized where total annual pasture utilization per hectare is maximized, rather than simply based on systems with exclusively pasture diets and the lowest per unit milk production costs. Consequently, the overall economic integrity of this model of milk production is predicated on high levels of pasture productivity allied to compact seasonal calving and a predominantly grazed pasture diet.

Foremost among the requirements of pasture-based production systems with a high level of reliance on grazed pasture, the capability to grow significant quantities (in excess of 10 t DM/hectare) of high-quality pasture predictably over a long grazing season is of paramount importance. In grazing dairy systems located in the temperate warm-summer or oceanic climate zones (New Zealand and Northern Europe), cool summer temperatures, moderate relative humidity, and evenly distributed precipitation during the year support predictable pasture production (Beck et al., 2018). Latitudinal positioning, continental and ocean current influences, and other factors result in different climates within temperate regions. Nonetheless, significant quantities of rainfall with an even seasonal distribution (approximately 750 mm with rain each month through the year) and low mean summer temperatures (<22°C) are valuable climatic characteristics for stable pasture production. Figure 1 illustrates mean monthly rainfall and air temperature for Ireland, Western France, Uruguay, and the South Island of New Zealand where pasture-based systems are commonplace based on suitable climatic conditions.

**Figure 1:**
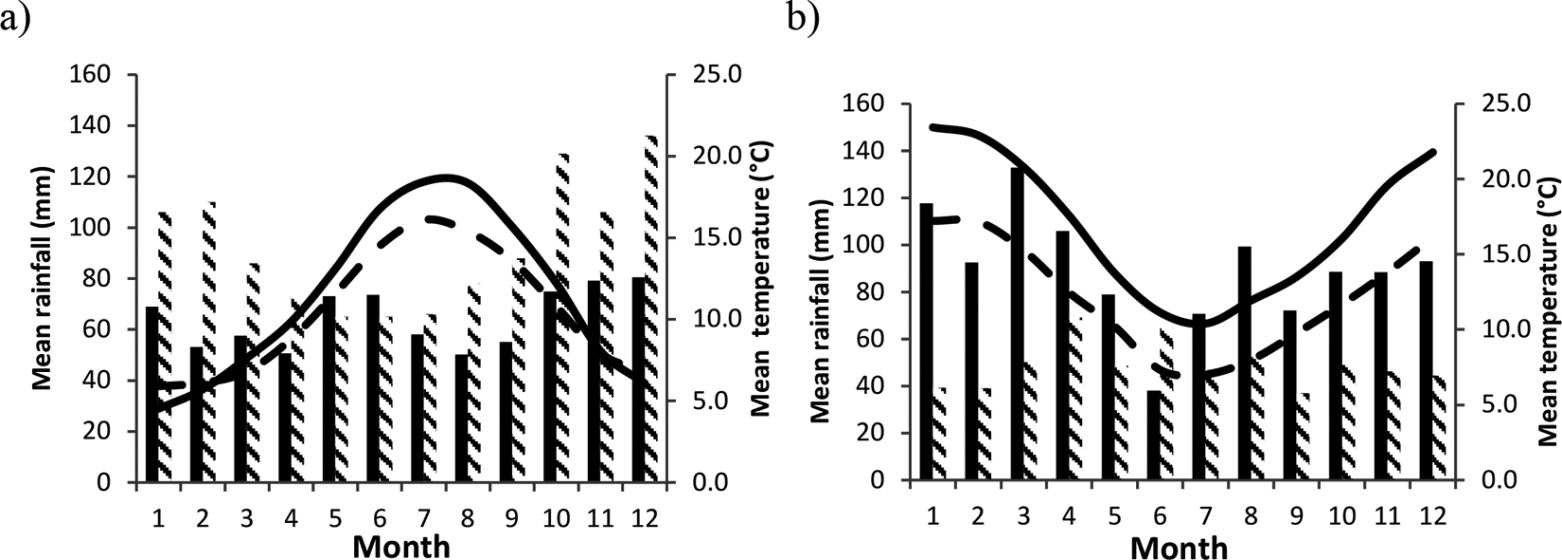
Seasonal variation in total monthly rainfall (columns; mm) and mean monthly air temperature (lines; °C) during the last 10 yr (2015–2024, inclusive) for (a) Northwest France (Le Pin, Normandy; black line and column) and southern Ireland (Teagasc, Moorepark, Co. Cork; dashed line and checkered column), and (b) Uruguay (INIA, La Estanzuela; black line and column) and New Zealand (Christchurch; dashed line and checkered column).

With the exception of some regions in the South Island of New Zealand where total rainfall is less than adequate for optimum grass production and irrigation is required, rainfall is distributed throughout the year in all locations. In contrast, the temperate hot summer or humid subtropical climate zones characterized by hot, humid summers and cool to mild winters define the pasture-based dairy systems from the Atlantic South American region (Uruguay, Northern Argentina, Southern Brazil). The high mid-summer mean air temperatures in these regions, in addition to maximum daily temperature typically exceeding 30°C, increase evapotranspiration inducing heat stress and plant dormancy, resulting in lower pasture growth (depending on rainfall) and reduced forage quality during summer. Looking to the future, requirements to reduce the use of both inorganic N and P fertilizers and irrigation water within all agricultural systems can be anticipated to reduce the control and increase variability in pasture growth patterns in all temperate regions. More frequent and intense periods of elevated mean and maximum summer temperatures and associated soil moisture deficits have been reported in most regions since the 1950s (IPCC 2023, 6th Assessment Report) and are anticipated to increase in intensity in the future (Perkins-Kirkpatrick and Gibson, 2017; Cera et al., 2025). Although the frequency and intensity of storms, heatwaves, and drought moisture deficits are expected to increase, these changes are expected to result in similar total pasture production but with increased pasture growth during autumn, winter, and spring and reduced growth during summer (IPCC 2023, 6th Assessment Report; Bateman et al., 2026). Consequently, pasture-based systems in temperate regions must be adapted to reduced summer pasture growth to facilitate additional pasture utilization and animal performance during the resurgent growth experienced when pasture growth resumes during autumn (Woodward et al., 2002; Fariña and Chilibroste, 2019; Lazzarini et al., 2019; Pedemonte et al., 2024).

The central importance of increased pasture utilization (t DM/ha) to the efficient performance of pasture-based dairy systems has been widely reported (Ramsbottom et al., 2015; Macdonald et al., 2017) based on achieving an alignment of animal feed requirements and pasture feed supply. Stocking rate (**SR**), which is generally defined as the number of animals allocated to an area of land (i.e., cows/ha), is a crucial factor that influences system efficiency in grazing dairy systems where pastures serve as the primary feed source (McMeekan and Walshe, 1963; McCarthy et al., 2011). Consequently, pasture-based systems are uniquely a compromise between the dual objectives of maximizing the utilization of pasture and maintaining high animal intakes and performance, with minimal use of supplemental feeds, mechanization, and capital infrastructure. Although it is always possible to have a very high stocking density at grazing that will maximize pasture utilization, when the feed demands of a high SR exceed the pasture production potential of the land area, increased levels of supplementary forages such as maize or grass silage or additional concentrate supplementation (or both) will be required to meet increased animal requirements, thereby inadvertently reducing the proportion of grazed pasture in the total individual animal diet (McCarthy et al., 2013). Consequently, the optimal SR should permit high levels of grazed pasture utilization while maintaining a long grazing season with a high proportion of grazed pasture in the animal diet (Macdonald et al., 2008). In that study, Macdonald et al. (2008) introduced the concept of comparative stocking rate (**CSR**), which was defined as kilograms of cow BW (at a standard BCS) per tonne of all feed DM available (kg BW/t DM offered). Their findings showed a quadratic relationship between CSR and profitability and suggested that the optimum CSR from a profitability perspective was observed at 78 kg BW/t DM available. Although CSR is a useful tool for comparing pasture-based systems, the need for estimates of pasture production is challenging due to inherent difficulties of accurately measuring pasture mass, highlighting the need for alternative metrics to compare and assess farm performance. On that basis, previous studies have recognized the number of grazing days per hectare per year achieved (calculated in this article as the number of grazing days at pasture without forage supplements multiplied by the SR on the grazing area) as the best indicator of pasture utilization, regardless of the method used to increase the SR (Hoden et al., 1991; Vérité and Delaby, 2000; McCarthy et al., 2011; Coffey et al., 2018).

Calving date and rate are also important determinants of feed utilization, feed quality, and milk production in pasture-based systems, through their impact on the alignment of feed demand and pasture feed supply. Pasture-based systems of milk production require rapid calving in spring to match feed supply and herd demand, while altering the mean calving date of the herd may have a role in reducing the reliance of such systems on purchased feeds, particularly at higher SR. The other main advantages of compact calving in spring are to allow additional DIM on high quality spring pasture and increased milk production to be achieved during the grazing season while aligning the nonlactation period during winter, when animal requirements are reduced and pasture growth is limited, to the use of conserved forages with lower feed quality (energy and protein content). In systems based on compact calving, grazed pasture-based feeding and low levels of supplementary feed, dairy cows with high fertility capacity in a short breeding period are required to utilize forages efficiently. By association, in situations where summer DM production is consistently and substantially reduced in temperate regions due to elevated temperatures and low moisture availability, Delaby et al. (2020) has proposed split compact calving (50% in spring, 50% in autumn) as a possibility to reduce summer feed requirements, while maintaining a high level of synchrony between pasture supply and demand over the full grazing season.

A fundamental tenet of efficient pasture-based systems involves using the cow to maximize and harvest most of the pasture grown, thereby minimizing supplemental feed and associated capital and labor costs (i.e., machinery for forage harvesting and slurry application, housing, and ancillary nongrazing costs). Systems based on grazed pasture intrinsically limit DMI compared with indoor and TMR diets (Kolver and Muller, 1998). Increasing pasture allowance to support higher levels of DMI also results in higher levels of feed refusals, decreased pasture utilization, and lower feed quality in subsequent feeding cycles (Pérez-Prieto and Delagarde, 2013; Delaby and Horan, 2017). Consequently, a unique feature of such systems is that grazing management and feed allocation decisions are tailored to optimize pasture growth, quality, and utilization by the grazing animal. The 2 most important aspects to routine grazing management practice which farmers must manage effectively for high productivity pasture-based systems are the grazing interval (i.e., when to graze, at the right pregrazing height) and the grazing intensity (i.e., how hard to graze, characterized by the postgrazing height). To consistently achieve high levels of pasture productivity on a long-term basis using grazing tolerant species, such as perennial ryegrass (*Lolium perenne*), the ideal average postgrazing residual is 4 to 5 cm, with each additional 1 cm of postgrazing height remaining above 4 cm corresponding to 250 kg DM/ha in wasted pasture, which will decay and contribute to reduced sward quality in subsequent rotations (McCarthy et al., 2011; Lee et al., 2013). Pregrazing height is the second routine management factor, and as perennial ryegrass is a “3-leaf” plant, delaying plant grazing until the third leaf emerges is important to maximize pasture production through leaf area and light interception by having the plant spend a greater proportion of its time in the most rapid phase of growth during leaf stage 3 (Fulkerson and Donaghy, 2001). Finally, and as part of grazing management, concentrate supplementation must be carefully managed within grazing systems. Although imported feeds can be used to support grazing systems during periods of feed shortage, they typically undermine profitability if used to increase overall feed demand (i.e., increase SR) because of the additional non-feed cost associated with marginal milk production, and so concentrate supplementation must be limited to maximize grass intake and pasture utilization. Both Ramsbottom et al. (2015) and Neal and Roche (2019) have previously reported that increased concentrate supplementation reduced the efficiency of grazing systems as evidenced by significant increases in total production costs (+€1.60 and €2.00 per €1.00 additional spend on feed) due to increases in both fixed and non-feed variable costs associated with reduced efficiency of supplement use in grazing systems.

Pasture-based systems have typically and traditionally been defined as those systems in which grazed pasture constitutes a significant portion of the total animal diet (García and Fulkerson, 2005). More recently, more specific additional definitions have been offered in literature ranging from the specific quantities of fresh or DM pasture in the lactation diet of dairy cows, or indeed relating to the length of time (in days) lactating animal spend outdoors in a paddock (O'Brien et al., 2018; NZMP, 2022). To confirm the specific dietary characteristics of temperate pasture-based grazing systems, we have reviewed the literature from recent full-lactation farm system experiments that have reported the proportion of grazed pasture in the total animal diet (on a DM basis) in addition to other key characteristics.

To be included in the database, the required experimental data included grazing platform area SR, grazing days per cow without forage supplements, concentrate supplementation (kg DM/cow), and the proportion of grazed pasture in the total annual dairy cow diet. In the case of partial grazing days where some forage supplementation occurred during the grazing season, only a half day of grazing was counted to adjust for the forage supplementation. The final database contained 56 experimental comparisons published between 2008 and 2024, with the majority of studies from Ireland, New Zealand, France, or Uruguay (and listed in Table 1). The average (±SD) experiment was undertaken at 2.76 (±0.707) cows per hectare, with 64% (±16.6%) grazed pasture in the total animal diet on a DM basis, average grazing days per cow without forage supplements of 239 (±53.9) d, and average concentrate supplementation of 814 (±892.0) kg DM/cow. Based on the data, the proportion of grazed pasture in the total animal diet was described using linear regression according to the effects of the number of grazing days achieved without forage supplementation per cow (GD in days), the SR applied on the grazing platform (SR; cows/ha), and the total level of concentrate supplementation (CC; kg DM/cow per year). The results of the linear regression indicate that the 3 parameters evaluated explain a large part of the variation in grazed pasture proportion (R^2^ = 0.86; residual SE = 6.39). The predicted effect of grazing days, SR, and concentrate feed level on the proportion of grazed pasture in the dairy cow diet was described according to the following equation:

**Table 1:** The relationship between stocking rate, cow grazing days without forage supplementation, and concentrate supplementation level on the proportion of pasture in the diet of grazing dairy cows within 56 diverse recent farmlet experiments^1^

Item									
Stocking rate (cows/ha)	2.0	2.0	2.0	2.5	2.5	2.5	3.0	3.0	3.0
Pasture-only grazing days (no./cow)	150	200	**250**	150	**200**	250	**165**	200	250
Pasture-only grazing days (no./ha)	300	400	**500**	375	**500**	625	**500**	600	750
Concentrate feed (kg DM/cow)									
0	52	63	**73**	52	**62**	72	**55**	61	72
500	49	59	**69**	48	**58**	69	**51**	58	68
1,000	45	55	**66**	44	**55**	65	**47**	54	64
1,500	41	51	**62**	40	**51**	61	**43**	50	60

1 Data derived from recent published literature from full-lactation farm systems documenting pasture proportion and system characteristics (and ranges) within temperate grazing systems (Macdonald et al., 2008; Delaby et al., 2009, 2018, 2024; McCarthy et al., 2016; Coffey et al., 2018; McClearn et al., 2019; Chapman et al., 2021; Stirling et al., 2021; Edwards et al., 2022; Cahill et al., 2023; Fenger et al., 2023; Jezequel et al., 2024; Murray et al., 2024; Ortega et al., 2024; Taube et al., 2024; Gil-Zibil et al, 2025; Menegazzi et al., 2025a,b). Bold values highlight different combinations of stocking rate and grazing days per cow to obtain 500 grazing days per hectare.

Proportion of grazed pasture in the diet (%) = 24.0 + 0.02075 × GD − 1.36 × SR − 0.00757 × CC.

The prediction equation indicates that a 100 d increase in grazing days, a 0.5 cows/ha increase in SR, and a 500 kg DM increase in concentrate supplementation per cow result in a +2%, −0.7%, and −4% change in pasture proportion in the total dairy cow diet, respectively. The relationships between the proportion of grazed pasture and grazing days per hectare (the combination of grazing season length without forage supplementation and SR) and concentrate supplementation level are illustrated in Table 1 and Figure 2. Based on the data reported in these studies, a pasture-based system where the majority of the diet is grazed pasture (>50% of the total animal diet on a DM basis) is only achieved within systems when concentrate supplementation is maintained at low levels (<1,000 kg DM/cow; or approximately 15% of total DM) and a large number of pasture-only grazing days per hectare of the grazing platform (>500 d) are achieved simultaneously.

**Figure 2:**
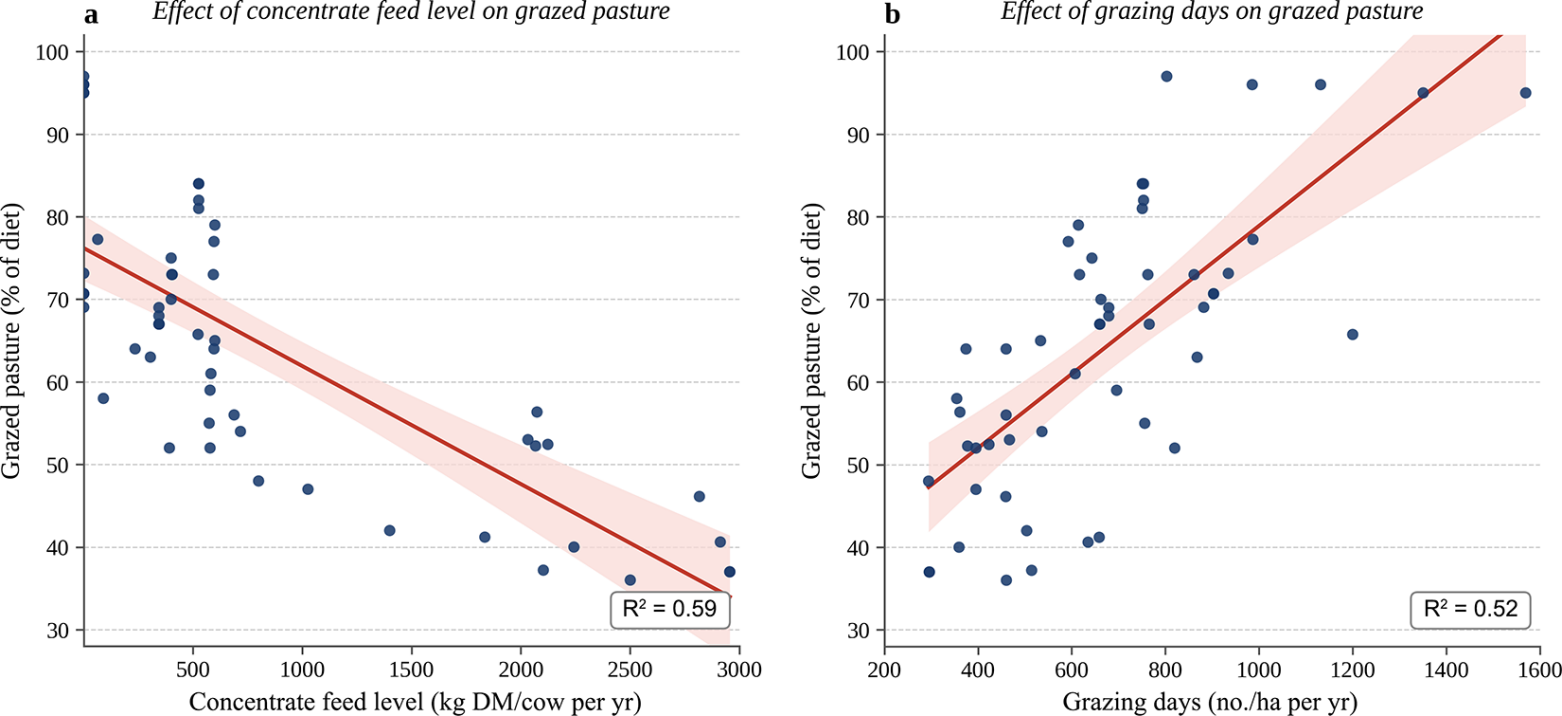
The relationship between (a) concentrate feed level (kg DM/cow) and (b) grazing days without supplement (d/ha), and the annual proportion of grazed pasture in dairy cow diets within temperate grazing systems. Solid red lines represent linear regressions, and shaded areas indicate the 95% CI around the fitted relationships.

An important consistent relationship was observed between concentrate feed level, grazing days per hectare without other non-pasture supplements, and grazed pasture proportion in dairy diets within our review of farm system studies. On that basis, we propose that a pasture-based dairy system can be defined as a dairy production system where the total diet of the dairy cow during the combined lactating and nonlactating period (equivalent to one complete year) contains a minimum of 50% of grazed pasture on a DM basis. Such systems are based on a large number of pasture-only grazing days per hectare on the grazing platform (>500 d) without other non-pasture supplements and with less than 15% of total animal feed requirements (equivalent to approximately 1,000 kg DM/cow) supplied from concentrate supplements. On that basis, we recommend that future grazing studies report these important criteria as critical defining characteristics of pasture-based grazing systems.

The results of this analysis across a range of recent farmlet system experiments within temperate regions illustrate a strong positive relationship between SR, grazing season length without supplements, concentrate feeding level per cow, and the proportion of grazed pasture achieved within pasture-based dairy systems. On that basis, these results support a more specific definition of a grazed pasture-based dairy system as a system where more than 50% of the total animal requirements (on a DM basis) are composed of grazed pasture, together with a maximum concentrate supplementation level of 15% of the total diet.
